# Structural Model for Factor X Inhibition of IgM and Complement-Mediated Neutralization of Adenovirus

**DOI:** 10.3390/v15061343

**Published:** 2023-06-09

**Authors:** Nicole Wagner, Dmitry M. Shayakhmetov, Phoebe L. Stewart

**Affiliations:** 1 Cleveland Center for Membrane and Structural Biology, Department of Pharmacology, Case Western Reserve University, Cleveland, OH 44106, USA; nxw243@case.edu; 2 Lowance Center for Human Immunology, Departments of Pediatrics and Medicine, Emory University School of Medicine, Atlanta, GA 30322, USA; dmitryshay@emory.edu; 3 Emory Vaccine Center, Emory University School of Medicine, Atlanta, GA 30322, USA; 4 Discovery and Developmental Therapeutics Program, Winship Cancer Institute of Emory University, Atlanta, GA 30322, USA

**Keywords:** adenovirus, neutralization, immunoglobulin M, complement C1, complement C3, complement C4, factor X, molecular dynamics

## Abstract

Adenovirus has strong therapeutic potential as an oncolytic virus and gene therapy vector. However, injecting human species C serotype 5 adenovirus, HAdv-C5, into the bloodstream leads to numerous interactions with plasma proteins that affect viral tropism and biodistribution, and can lead to potent immune responses and viral neutralization. The HAdv/factor X (FX) interaction facilitates highly efficient liver transduction and protects virus particles from complement-mediated neutralization after intravenous delivery. Ablating the FX interaction site on the HAdv-C5 capsid leaves the virus susceptible to neutralization by natural IgM followed by activation of the complement cascade and covalent binding of complement components C4b and C3b to the viral capsid. Here we present structural models for IgM and complement components C1, C4b, and C3b in complex with HAdv-C5. Molecular dynamics simulations indicate that when C3b binds near the vertex, multiple stabilizing interactions can be formed between C3b, penton base, and fiber. These interactions may stabilize the vertex region of the capsid and prevent release of the virally encoded membrane lytic factor, protein VI, which is packaged inside of the viral capsid, thus effectively neutralizing the virus. In a situation where FX and IgM are competing for binding to the capsid, IgM may not be able to form a bent conformation in which most of its Fab arms interact with the capsid. Our structural modeling of the competitive interaction of FX and IgM with HAdv-C5 allows us to propose a mechanistic model for FX inhibition of IgM-mediated virus neutralization. According to this model, although IgM may bind to the capsid, in the presence of FX it will likely retain a planar conformation and thus be unable to promote activation of the complement cascade at the virus surface.

## 1. Introduction

Various serotypes of human adenovirus (HAdv) are appealing vector platforms for intravenous delivery of gene therapies, as well as emerging vectors for oncolytic systemic virotherapy in cancer patients [1,2,3,4]. However, systemic delivery of therapeutics via adenoviral vectors is challenged by the host’s immune responses. Following intravenous injection, bloodborne HAdv will encounter a variety of host proteins, including the antibody immunoglobulin M (IgM), complement proteins, and coagulation factors such as factor X (FX) [5]. Complement proteins neutralize HAdv by “sealing” it [6,7] and blocking release of the virally encoded membrane-lytic protein VI [8], which is required for entry of the virus into the cytosol of host cells. A thorough understanding of the structural basis for interactions between HAdv-C5 and host immune proteins would be advantageous in the engineering of therapeutic adenoviral vectors.

Notably, viruses can be recognized by natural IgMs, which exist prior to antigen exposure [9]. An initially planar IgM binds to the viral surface, where it adopts a staple-shaped or bent conformation that is recognized by complement [10]. IgM, or IgG, binding to the virus marks the beginning of the classical pathway of the complement cascade, an innate immune response that occurs immediately upon infection in order to defend the host against viruses and other pathogens [11,12]. The C1 complex, which is composed of complement proteins C1q, C1s, and C1r, binds to the bent conformation of IgM. The critical residues in the IgM Fc region that are involved in the interaction with C1q [13] are only exposed in the bent IgM conformation [14], preventing premature activation of the complement cascade. Binding of C1q to the viral surface activates the proenzymes C1s and C1r in the C1 complex. The C1 proteases in turn cleave complement component C4 into C4b and complement component C2 into C2b, forming the classical C3 convertase (C4b2b, formerly known as C4b2a) [12]. C4b has a reactive thioester that can covalently bind to the viral surface.

After C4b is bound and the classical C3 convertase (C4b2b) is formed, the convertase is able to cleave complement component C3 into C3b. C3b is composed of a shorter N-terminal β-chain connected by a disulfide bond to the longer C-terminal α-chain, which contains the thioester domain [15]. The thioester of C3b is formed by residues Cys1010 and Gln1013 and, like the thioester of C4b, is able to covalently bind to the viral surface [16]. Subsequently, the alternative C3 convertase, formed by C3b and factor B (C3bBb), can facilitate an amplification loop, which leads to additional C3b binding to the virus. Various complement regulators in the blood dampen the amplification loop to help protect the host from off-target complement-mediated attack. Factor H is the primary negative regulator of the C3 amplification loop in the fluid phase [17].

In addition to IgM and complement proteins, blood coagulation factors also bind HAdv and influence its tropism [18]. Coagulation factor X (FX) binds to the hexon proteins of the viral capsid with picomolar affinity [19,20,21]. This is in contrast to IgM, which binds via high avidity rather than affinity, due to its multivalent interaction with the capsid surface through its Fab arms [5]. Binding of FX to HAdv triggers an innate immune response via the TLR/NF-κB pathway and leads to efficient liver transduction of the virus but blocks natural antibodies and complement proteins from neutralizing the virus [22,23]. The key hexon residue involved in FX binding, T425, can be mutated to alanine to ablate FX binding to HAdv [19].

In recent studies conducted with mouse serum, natural IgM was able to inactivate an FX-binding ablated HAdv-C5 mutant (termed Ad5-FX*) but not the wild-type form of the virus (Ad5-WT) [24]. Compared to Ad5-WT, Ad5-FX* showed increased deposition of complement proteins C3b and C4b on the capsid surface, as well as an increased amount of C3b relative to C4b (C4b:C3b ratio ~1:1.5). Although it is evident that FX binding protects adenovirus from complement-mediated neutralization, the structural basis of how FX and IgM in the bloodstream compete for access to the capsid surface—and how FX can ultimately “win” this competition—is unknown.

In this study, we used molecular modeling and molecular dynamics (MD) simulations to explore a structural basis for FX inhibition of IgM- and complement-mediated neutralization of adenovirus. We assembled a model of an Ad5-FX* facet consisting of 18 hexon trimers, three penton base pentamers, and three fibers. We then placed a bent IgM in a variety of positions and modeled subsequent recruitment of C1 and C4b, as well as amplification of C3b binding to the facet. Our MD simulations indicate that, when C3b is bound at a vertex of the facet near a penton base and fiber, C3b can form multiple stabilizing interactions with the penton base Arg-Gly-Asp (RGD) containing loops, as well as with fiber. We propose that these interactions would effectively “seal” and neutralize the virus by blocking release of membrane lytic protein VI. We also constructed models to show how decoration of the Ad5-WT facet with FX may allow planar IgM to bind to the viral surface via a few Fab arms but prevent the conformational change of IgM to the bent state. If the bent conformation of IgM cannot form on the viral surface, then recruitment of complement proteins would be blocked. Even if a bent IgM is occasionally able to form on the viral surface in the presence of FX, our models indicate that surrounding FX molecules may effectively crowd out C4b and C3b binding to the capsid. These models enhance our understanding of the varied host immune responses that adenoviral vectors may encounter.

## 2. Materials and Methods

### 2.1. Initial Model Building

Coordinates for 18 hexon trimers representing one triangular facet were selected from the cryoEM HAdv-C5 coordinates (PDB: 6B1T) [25] using UCSF ChimeraX v1.5 [26]. Technically only 12 hexon trimers comprise an icosahedral facet of HAdv-C5, but six additional hexon trimers were included along the facet edges in order to form a complete triangular array. Missing HVR-1 loops were filled in for each of the 54 hexon monomeric subunits using SWISS-MODEL [27]. Coordinates for three penton base pentamers were also selected from 6B1T in ChimeraX v1.5 and their RGD loops filled in with SWISS-MODEL, resulting in different RGD loop conformations for each of the subunits in a given pentamer. A penton base pentamer was added to each vertex of the facet.

The structure of the C-terminal knob domain and last four shaft repeats of the homologous HAdv-C2 fiber were obtained from PDB: 1QIU [28]. The first 17 shaft repeats were initially modeled with coordinates from residues 361–376 added onto the N-terminal end of the shaft using UCSF Chimera v1.16 [29]. Additions to the shaft were translated and rotated by increments of 13 Å and 51.4° per repeat. The coordinates of the N-terminal tails of fiber were obtained from PDB: 3IZO [30]. For each chain of the fiber, SWISS-MODEL was used with the initial model as a template to generate a more realistic model with the correct fiber sequence and without any gaps. Fiber chains were positioned on penton base pentamers by alignment with their corresponding N-terminal tails in PDB: 3IZO using the Matchmaker tool in ChimeraX v1.5.

A model of the pentameric IgM in a bent conformation was constructed from structures of mouse IgM Fc domains Cµ2 (PDB: 4JVU), Cµ3 (PDB: 4BA8), and Cµ4 (PDB: 4JVW) [31], and human IgM Fab fragment (PDB: 1DEE) [32]. Domains were positioned into the cryoET subtomogram average density of the pentameric IgM-C1-C4b_2_ complex on antigen-bearing lipid membranes (EMD-4945) [14]. The bent IgM model was placed in four different starting positions on the facet, with the same pair of Fab arms placed over each of the four hexon trimers in a single asymmetric unit. From each starting position, IgM was then rotated by 90°, 180°, and 270° about its Fc platform, resulting in a total of 16 IgM positions.

The initial model of the C1 complex, comprising the C1q hexamer and the C1r_2_s_2_ tetrameric protease, was derived from SAXS data [33]. Modifications were made to the C1q coordinates of this model for improved agreement with cryoET density of pentameric-IgM-C1-C4b_2_ complex (EMD-4945). The C1r coordinates were replaced with domains from the human C1rC1s complex (PDB: 6F1C) [34] and the human C1r catalytic domain (PDB: 1GPZ) [35] and the C1s coordinates were replaced with domains from the human C1rC1s complex (PDB: 6F1C) and the human C1s zymogen (PDB: 4J1Y) [36]. To position C1 on the bent IgM model, coordinates of the domains were positioned into the subtomogram average density of the IgM-C1-C4b_2_ complex (EMD-4945). The C1q subunit positioned over the gap in the IgM Fc platform was bent upward such that its globular domain hovers over the facet.

Coordinates of complement C4b were obtained from the crystal structure PDB: 5JTW [37]. The subtomogram average density of the IgM-C1-C4b_2_ complex (EMD-4945) and figures from the corresponding reference [14] were used to guide the positioning of two C4b molecules relative to IgM and the C1s domains of C1. Four of the 16 IgM positions we modeled have only one C4b, as the position of the second C4b would result in its reactive thioester hanging over the edge of the facet and therefore being unable to bind an amino or hydroxyl group on the represented facet.

### 2.2. Modeling Addition of C3b to the Facet

Coordinates of complement C3b were obtained from the crystal structure PDB: 5FO7 [38]. Addition of C3b molecules was performed according to a convertase-substrate model using the structure of cobra venom factor (CVF) in complex with complement C5 (PDB: 3PVM) [39]. First, the CVF of 3PVM was aligned with our positioned C4b using the Matchmaker tool in UCSF ChimeraX v1.5. The coordinates of 5FO7, representing the incoming C3, were then aligned with the C5 of 3PVM using Matchmaker. The C3b was then translated up to ~300 Å from C4b [40] (all the way across the facet surface) or until further movement was obstructed by another molecule (e.g., the HAdv-C5 fiber or a Fab arm of IgM). C3b was translated and rotated such that its thioester domain was positioned near the facet surface. Additionally, C3b was rotated as needed to expose its binding interface for the next incoming C3. The CVF of 3PVM was then aligned with the first C3b, and a new copy of the 5FO7 coordinates was aligned with C5 of 3PVM, thereby using the convertase-substrate model again for addition of a second C3b in cases where there was ample nearby space. This process was repeated until there was a maximum of three C3b molecules bound per facet. In some cases, there was space on the facet for additional copies of C3b, but we chose an arbitrary maximum to represent negative regulation of the amplification loop.

### 2.3. Modeling Competition between FX and IgM on HAdv-C5

Model coordinates for the zymogenic form of FX [41] were positioned relative to hexons within the HAdv-C5 facet as observed in a cryoEM structure of the FX/HAdv-C5 complex [19]. Specifically, the FX-GLA domain was placed in the central depression of each hexon trimer. In the cryoEM structure of the FX-HAdv-C5 complex, the best-resolved FX density was observed above the hexon trimers adjacent to the icosahedral threefold symmetry axis in the center of the facet. Therefore, in our model we positioned copies of FX in these three hexon trimers to resemble the cryoEM density at these locations. For the remaining fifteen hexon trimers in the model facet, we positioned FX in a more random manner to account for the domain flexibility of FX and the more diffuse FX density observed above the remaining hexons in the cryoEM structure.

The bent IgM model was placed onto the FX-loaded facet as in the first of our 16 IgM positions (with a pair of Fab arms over hexon trimer #1 in the asymmetric unit and no rotation about the Fc platform). The Clashes tool in UCSF ChimeraX v1.5 was used to identify FX molecules that clashed with IgM, and any clashing FX molecules were removed. A planar IgM model was prepared by straightening the Fab arms of the bent IgM model with UCSF Chimera v1.16. The planar IgM model was positioned with the same pair of Fab arms located approximately over hexon trimer #1. The Clashes tool in UCSF ChimeraX v1.5 was again used to identify FX molecules clashing with the IgM, and these FX molecules were subsequently removed.

### 2.4. Molecular Dynamics Simulations

Molecular dynamics simulations were performed to assess the ability of C3b to form energetically favorable interactions with multiple RGD loops of the penton base or with the fiber and at least one RGD loop. We generated 11 starting models of facet vertices (the penton base pentamer and trimeric fiber) with a single C3b positioned nearby. The model of the HAdv-C5 penton base was prepared as discussed under “Initial model building” above. The model of the HAdv-C5 fiber was also prepared as discussed under “Initial model building” above, with an additional peptide containing N-terminal amino acids 1–6 added for each chain using the Build Structure tool in UCSF Chimera v1.16. Coordinates of complement C3b were obtained from PDB: 5FO7 with SWISS-MODEL used to fill in missing internal loops. Peptides containing C3b amino acids 666–667 and 749–751 were added at chain termini using the Build Structure tool.

MD simulations were performed with NAMD v2.14 [42] on the Case Western Reserve University (CWRU) high-performance computing (HPC) cluster. The system was solvated in a water box and minimized for 20 ps. The simulation was then run at a constant temperature of 310 K for 2 ns using the Chemistry at Harvard Molecular Mechanics (CHARMM) force field [43]. Atoms within the base portion of penton base (amino acids 38–296 and 377–571) were held fixed; all other atoms, including those in penton base RGD loops, fiber, and C3b, were permitted to move during the simulation.

### 2.5. Calculation of Non-Bonded Interaction Energies

For the ending coordinates of the MD simulations, nonbonded interaction energies (comprising the sum of the van der Waals and electrostatic contributions) were calculated between C3b and the HAdv-C5 fiber, and between C3b and each chain of the penton base individually. Combinations with nonzero interaction energies were further broken down to determine contributions of the α- and β-chains of C3b, contribution of the thioester domain of C3b, and the contribution of the penton base RGD loops. NAMD v2.14 and the NAMD Energy plugin of VMD v1.9.4 [44] were used to calculate the interaction energies between chains and defined domains. In addition, pairwise residue interaction energies between C3b α-chain residues 990–1290 (thioester domain) and penton base residues 297–376 (RGD loop) were calculated for the final frame of the MD trajectories using gRINN v1.1.0.hf1 [45].

## 3. Results

### 3.1. Modeling Natural IgM, Complement C1, and C4b Binding to HAdv-C5

**Figure 1:**
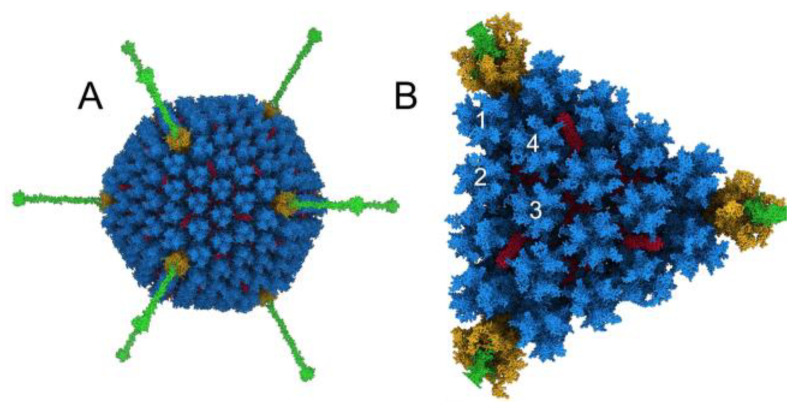
Adenovirus capsid structure. (**A**) Structure of the HAdv-C5 capsid viewed along an icosahedral threefold symmetry axis. Hexon trimers are depicted in blue, hexon-interlacing proteins (protein IX) in red, penton base in gold, and fibers in green. The fibers were modeled based on PDB entries 1QIU [28] and 3IZO [30]; the rest of the capsid was adapted from PDB: 6B1T [25]. Only alpha carbons are shown. (**B**) All-atom representation of a single facet of the capsid, viewed looking down an icosahedral threefold symmetry axis. Proteins are colored as in panel A. Four unique hexon positions within a single asymmetric unit are numbered 1–4.

HAdv-C5 has a striking icosahedral shape with prominent fibers protruding from the vertices (Figure 1A). The diameter of the capsid is ~926 Å [46], not including the fibers, and the full-length modeled fibers are ~350 Å long. The hexon proteins form the majority of the capsid, with penton base and fiber at the vertices, and the hexon-interlacing protein, protein IX, stabilizing the hexon portion of the capsid [25]. These four capsid proteins, hexon, penton base, fiber and protein IX, are accessible on the outer surface of the HAdv-C5 capsid. They provide possible interaction sites for host factors that the virus is exposed to within the blood after intravenous delivery of adenovirus-based vectors or upon natural disseminated adenovirus infections. Previous studies have shown that natural IgMs bind HAdv-C5 via the flexible hexon hypervariable region 1 (HVR1) loop exposed at the surface of the capsid [24]. Twelve copies of the trimeric hexon comprise each facet of the HAdv-C5 capsid, with four unique hexon positions within the icosahedral asymmetric unit (Figure 1B). In order to form a complete triangular array with four hexon trimers along each edge of the facet, a total of 18 hexon trimers were included in the modeled facet. Although we built a model of the WT HAdv-C5 facet, it is also a reasonable stand-in for Ad5-FX*, which has a point mutation in hexon (T425A) to ablate FX binding.

**Figure 2:**
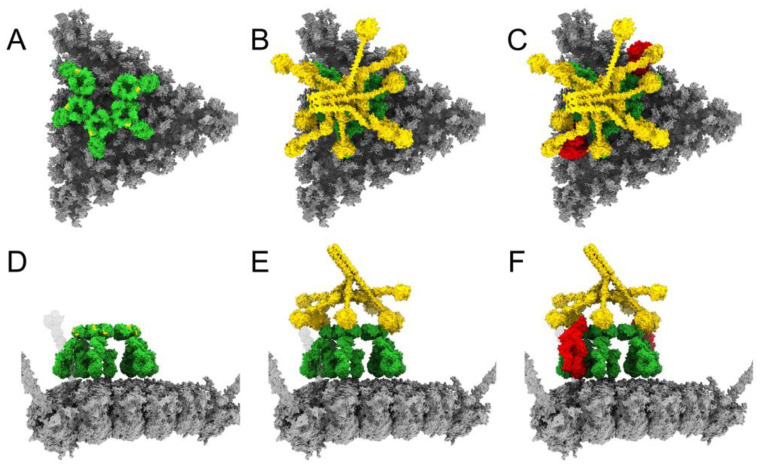
Adenovirus facet with modeled IgM, C1, and C4b. (**A**) HAdv-C5 facet (gray) shown with bent IgM bound (green). The DLPSP motifs on IgM, required for mediating C1q binding [13], are colored gold. The pair of Fab arms counterclockwise from the gap in the Fc platform are placed over hexon trimer #1 in the asymmetric unit. The Fab arms of IgM bind HVR1 loops of the hexon proteins [24], which are exposed at the top of the facet. (**B**) The complement C1 complex (gold), consisting of the C1q hexamer and the C1r_2_s_2_ tetrameric protease, binds to IgM. The C1q subunit over the gap in the Fc platform is bent upward with its globular domain hovering above the facet. (**C**) Two copies of complement C4b (red) are positioned adjacent to IgM and underneath the interacting C1q and C1s subunits as observed in the pentameric-IgM-C1-C4b_2_ complex [14]. (**D**) Side view of panel A resulting from a 90° rotation, showing more of the gold-colored DLPSP motifs on the otherwise green IgM. (**E**) Side view of panel B. (**F**) Side view of panel C.

When modeling the interaction of IgM with Hadv-C5 or Ad5-FX*, we used a bent, or dome-shaped, model of pentameric IgM based on cryoelectron tomography (cryoET) structures of IgM, C1, and C4b complexes on antigen-bearing lipid membranes [14]. The bent model of IgM has ten Fab arms poised to interact with antigens on a relatively flat surface. Therefore, we reasoned that IgM would most likely bind to a facet of the HAdv-C5 capsid, rather than straddle hexons on either side of a capsid edge. Positioning a bent IgM over an HAdv-C5 facet shows that there is ample room for one IgM to bind to a facet, but probably not enough room for two IgMs (Figure 2A,D). The bent conformation of IgM exposes the DLPSP (Asp-Leu-Pro-Ser-Pro) residues that are critical for mediating C1q binding [13]. Using the cryoET density of the pentameric-IgM-C1-C4b_2_ complex (EMD-4945) [14] as a guide, we positioned a model for the C1 complex over the modeled IgM (Figure 2B,E) and added two copies of C4b (Figure 2C,F). The reactive thioester of C4b binds covalently to nearby hydroxyl or amine groups [47], of which there are many on the facet surface.

### 3.2. Complement C3b Covalent Binding and Amplification

Having positioned C4b molecules on the facet, we proceeded to add copies of C3b to the facet model. The crystal structure of C4b is remarkably similar to that of its paralogue C3b, suggesting that the C3 convertases C4b2b and C3bBb share a similar architecture and mode of substrate interaction [48]. The interaction between a bound C4b (or bound C3b) molecule and an incoming C3 molecule can be modeled based on the structure of cobra venom factor (CVF) in complex with complement C5 [39]. Both C4 and C3 undergo similar conformational changes after they are cleaved to form C4b and C3b [48]. The reactive thioester groups of C4b and C3b are activated during the cleavage and conformational change process. While the reactive thioester of C4b covalently binds to either hydroxyl or amine groups, C3b preferentially binds to hydroxyl groups [47].

**Figure 3:**
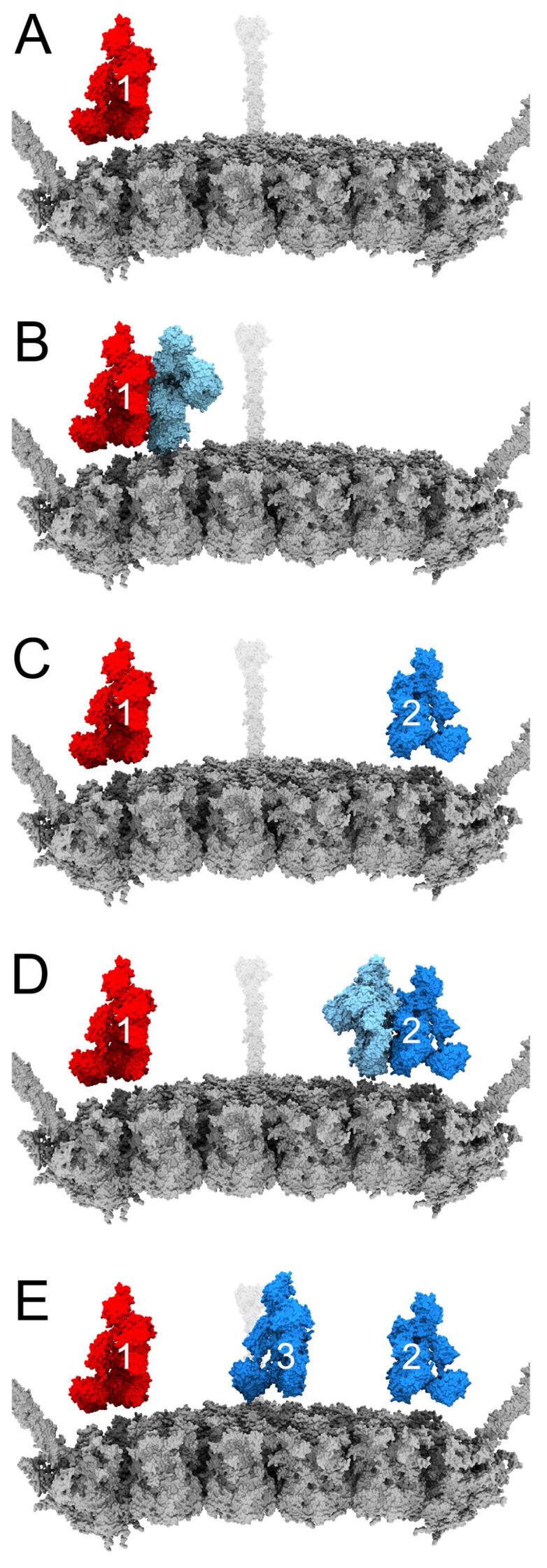
Model spacing of C4b and C3b on an adenovirus facet. C4b and C3b copies are numbered in the order of addition. (**A**) Side view of a facet (gray) with a covalently bound C4b (red) at one end, near a penton base with protruding fiber. (**B**) The covalently bound C4b interacts with the substrate, C3 (light blue). C4b and C3 are aligned according to the C5-cobra venom factor (CVF) complex (PDB: 3PVM) [39]. The protease subunit C2b of the C3 convertase (C4b2b) is not shown. (**C**) Facet with covalently bound C4b together with a covalently bound C3b (blue) positioned ~300 Å away. (**D**) Facet with covalently bound C4b and covalently bound C3b, with C3b interacting with another substrate C3 molecule. C3b and C3 are aligned according to the C5-CVF complex (PDB: 3PVM) [39]. The protease subunit Bb of the C3 convertase (C3bBb) is not shown. (**E**) Facet with one covalently bound C4b and two covalently bound C3b molecules.

Modeling of C3b on the adenovirus facet begins with a single C4b bound to the facet (Figure 3A). C4b, together with C2 and the action of nearby C1 proteases, forms the C3 convertase (C4b2b) [12]. The first incoming copy of C3 interacts with the substrate interaction side of C4b (Figure 3B) and is cleaved by the C3 convertase to form C3b. The reactive C3b intermediate has a minimum lifetime of ~60 µs, during which time it can diffuse up to 300 Å away from the site where it was activated [40]. Given that the side length of the facet is also approximately 300 Å, we chose to show C3b traveling the full distance across the facet from the C4b interface (Figure 3C) unless its path was obstructed by another molecule. We also anticipated that C3b was free to rotate as it diffused above the capsid surface before binding.

After positioning the first C3b on the facet, we continued to use the convertase-substrate model [39] to model the amplification of C3b binding to adenovirus. The C3b-C3 interface of the C3bBb convertase is presumed to be the same as the C4b-C3 interface, so we added C3b copy *n* at the binding interface of C3b copy *n*−1 if the interface was unobstructed (Figure 3D), then translated copy *n* (Figure 3E). Binding positions for C3b were considered feasible if the reactive thioester, located at the underside of its thioester domain, was able to contact the facet surface. We limited the number of C3b molecules per facet to a maximum of three to account for the negative regulation of the amplification loop.

**Figure 4:**
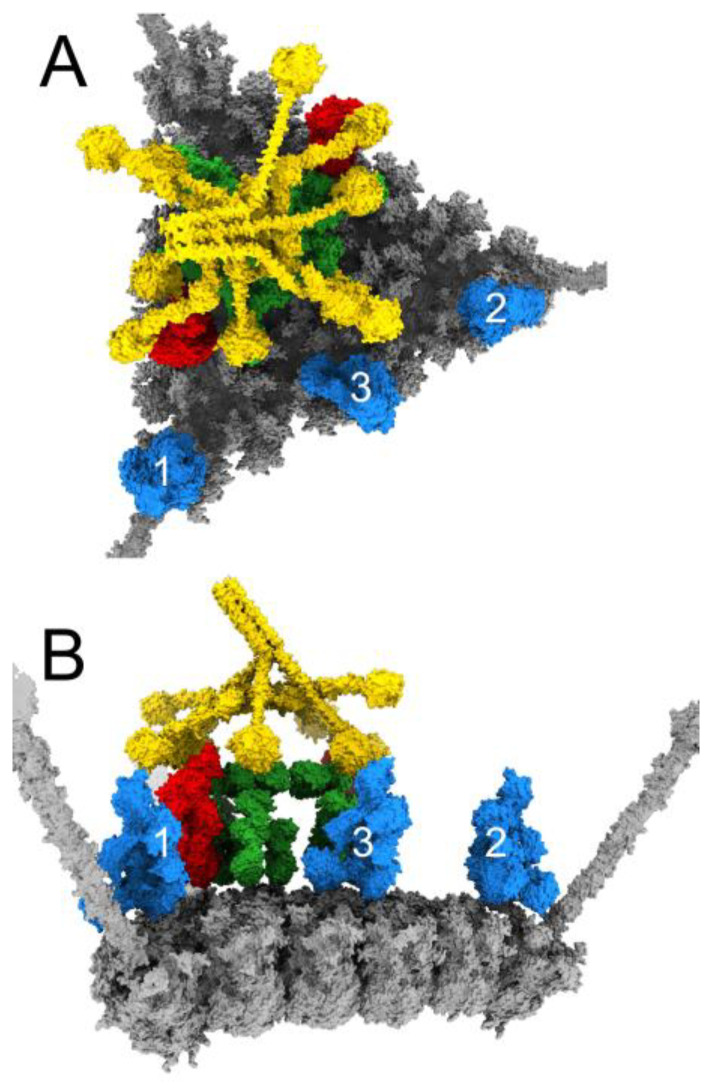
Adenovirus facet with modeled IgM, C1, C4b, and C3b. (**A**) HAdv-C5 facet (gray) shown with bent IgM (green), complement C1 (gold), two copies of complement C4b (red), and three copies of complement C3b (blue). The positions of IgM, C1, and C4b are the same as in Figure 2. The C3b copies are numbered in the order they were added. The first copy of C3b was aligned to the interface on C4b following the template of the C5-CVF complex [39], then translated until blocked by the fiber. The second copy of C3b was aligned to the interface of the first C3b and translated ~300 Å across the facet. The third C3b was added onto the interface of the second C3b and translated halfway back across the facet. (**B**) Side view of panel A resulting from a 90° rotation.

**Figure 5:**
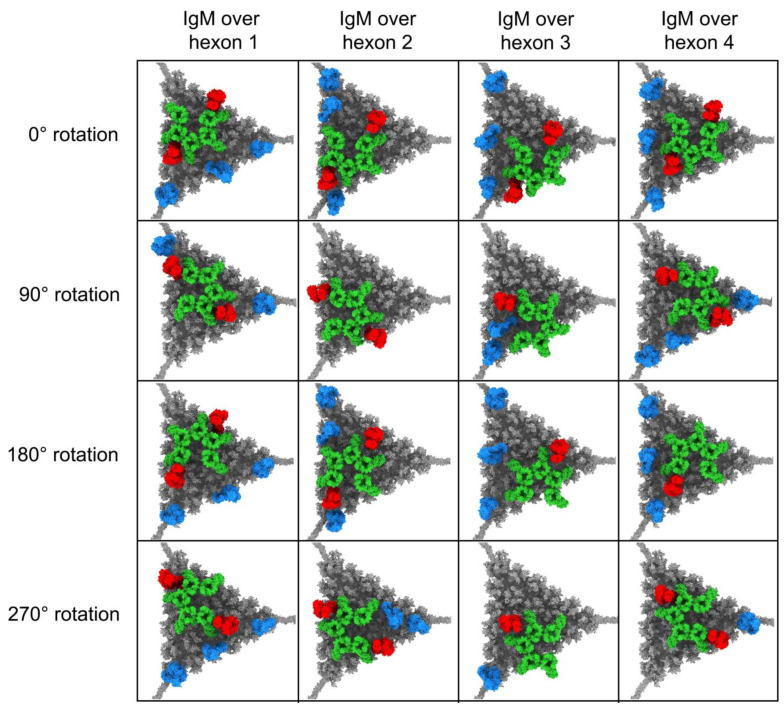
Adenovirus facet models with 16 poses of IgM, with covalently bound C4b and C3b. IgM was placed in four initial poses (considered “0° rotation” and shown in the top row), each with the pair of Fab arms counterclockwise from the gap in the Fc platform placed over a different hexon trimer (# 1, 2, 3, or 4) in the asymmetric unit. The models shown in the next three rows were prepared by rotating IgM 90°, 180°, or 270° about the Fc platform from its starting pose at 0° rotation. The positioning of IgM on the facet determines the placement of C1 (not shown) and C4b, and affects the possible binding sites for C3b, resulting in different numbers of covalently bound C4b and C3b molecules for each facet/IgM model.

As a representative example, Figure 4 shows the facet model that started with a pair of IgM Fab arms positioned over hexon one in the asymmetric unit (as shown in Figure 2). In this model, one C4b copy has led to recruitment of three C3b copies, while the other C4b was unable to recruit an initial C3 to the facet due to its position close to the facet edge. Since the position of IgM on the facet seems to affect the amplification of C3 and the number of C3b molecules that can bind to the facet, we built a total of 16 different facet/IgM models (Figure 5). In these various facet/IgM models, IgM is positioned over each of the 4 hexons in the asymmetric unit and rotated by 0°, 90°, 180°, or 270° with respect to the IgM position in the first model. We added C4b and C3b molecules to each facet/IgM model following the same approach as used for the first model. Of our 16 IgM starting positions, 11 have a copy of C3b at two vertices of the facet. Only one starting IgM position resulted in both C4b copies being unable to recruit a first C3b. This was a consequence of both C4b molecules being bound near the facet edge. The remaining four models result in a copy of C3b at one vertex of the facet (Figure 5).

### 3.3. Molecular Dynamics Simulations with HAdv-C5 Penton Base, Fiber, and C3b

**Figure 6:**
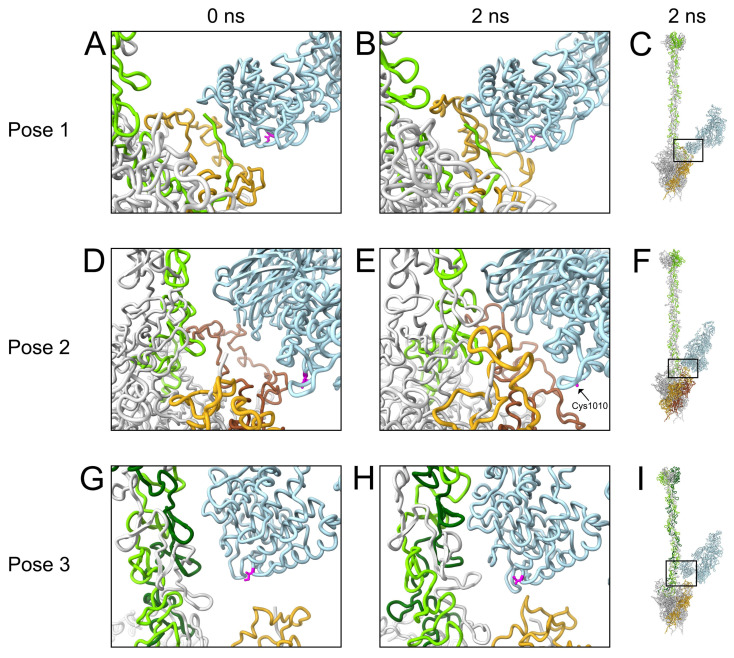
MD analysis of C3b interacting at the adenovirus vertex. (**A**) Starting coordinates (0 ns) of the MD trajectory for C3b pose 1. C3b is in light blue; penton base chain P2 is in gold; fiber chain F2 is in light green. Residue Cys1010 of C3b is in magenta. (**B**) Ending coordinates (2 ns) of the MD trajectory for C3b pose 1. (**C**) Zoomed-out view of the ending coordinates for C3b pose 1, showing the entire penton base, fiber, and C3b. The approximate region shown in panel B is enclosed in a box. (**D**) Starting coordinates (0 ns) of the MD trajectory for C3b pose 2. Penton base chain P1 is in gold; penton base chain P2 is in brown; fiber chain F3 is in light green. (**E**) Ending coordinates (2 ns) of the MD trajectory for C3b pose 2. (**F**) Zoomed-out view of the ending coordinates for C3b pose 2. (**G**) Starting coordinates (0 ns) of the MD trajectory for C3b pose 3. Penton base chain P4 is in gold; fiber chain F2 is in light green; fiber chain F3 is in dark green. (**H**) Ending coordinates (2 ns) of the MD trajectory for C3b pose 3. (**I**) Zoomed-out view of the ending coordinates for C3b pose 3.

As described in Section 3.2 above, most of our models contain C3b copies near two vertices, and all but one model contains a C3b near at least one vertex. We hypothesized that the presence of C3b near a vertex could lead to multiple stabilizing interactions between C3b and the penton base and fiber, essentially sealing the virus shut and preventing release of membrane lytic protein VI from the capsid. In particular, we anticipated that C3b would be able to entangle multiple RGD loops of the penton base. In order to test our hypothesis, we prepared 11 sets of starting coordinates for vertices containing one copy of C3b, the penton base pentamer, and the fiber, solvated in water. C3b was positioned with its thioester group, formed by Cys1010 and Gln1013, near the vertex proteins. The five penton base chains are denoted as P1-P5, and the three fiber chains as F1-F3. The coordinates of the penton base pentamer were held fixed except for the flexible RGD loops. After running 2 ns MD simulations, we evaluated the stabilizing nonbonded interaction energies between C3b and each individual penton base chain, and between C3b and the fiber. C3b in 3 of the 11 vertex models showed extensive interactions with penton base and fiber at the end of the MD simulation (Figure 6).

Figure 6A,D,G show these three starting coordinate sets, each with C3b positioned in a different orientation with respect to the vertex. Figure 6B,E,H show the corresponding ending coordinates after 2 ns simulations. Table 1 lists the nonbonded interaction energies calculated for the ending coordinates shown in Figure 6.

At the end of the C3b pose 1 trajectory, C3b has formed strong nonbonded interactions with chain P2 (−305 kcal/mol, with 93% from electrostatics and 7% from Van der Waals or VdW interactions) and chain F2 (−198 kcal/mol, 99% electrostatics, 1% VdW). From a zoomed-in perspective, comparison of the starting and ending coordinates (Figure 6A,B) indicates that the RGD loop of chain P2 has moved closer to the thioester domain of C3b. It also appears that the interaction with chain F2 occurs primarily via the N-terminal tail of the fiber.

At the end of the C3b pose 2 trajectory, C3b has formed strong nonbonded interactions with chains P1 (−58 kcal/mol, 78% electrostatics, 22% VdW), P2 (−52 kcal/mol, 100% electrostatics), and F3 (−173 kcal/mol, 100% electrostatics). Comparing zoomed-in views of the starting and ending coordinates (Figure 6D,E), it is apparent that the RGD loops of chains P1 and P2, as well as chain F3, have shifted closer to C3b over the course of the trajectory.

At the end of the C3b pose 3 trajectory, C3b has formed strong nonbonded interactions with chains P4 (−75 kcal/mol, 93% electrostatics, 7% VdW), F2 (−123 kcal/mol, 95% electrostatics, 5% VdW), and F3 (−47 kcal/mol, 79% electrostatics, 21% VdW). Comparing zoomed-in views of the starting and ending coordinates (Figure 6G,H), it appears that the RGD loop of chain P4, as well as chains F2 and F3, have all moved closer to the thioester domain of C3b.

In all three MD poses discussed, interactions of C3b with the penton base occurred entirely through the RGD loops, with no energy contributed by interactions between C3b and other regions of the penton base. For the most part, interactions with the fiber and RGD loops occurred via the thioester domain of the C3b α-chain, with the remainder of the α-chain and the shorter β-chain contributing no nonbonded interaction energy. Key exceptions are found in pose 2, where the β-chain of C3b mediates interactions with penton base chain P2 and fiber chain F3, while the C3b thioester domain mediates the interaction with penton base chain P1. The nonbonded interaction energies are similar for the C3b thioester domain/chain P1 interaction and the C3b β-chain/chain P2 interaction (−58 and −52 kcal/mol, respectively). The exceptions observed for pose 2 can be explained by its starting coordinates, where both C3b chains are in relatively close proximity to the penton base and fiber. In comparison, the starting coordinates of poses 1 and 3 place only the C3b thioester domain within range to establish interactions with the vertex proteins. The nonbonded interaction energies of the C3b β-chain with chains P2 and F3 in pose 2 are entirely electrostatic. In comparison, interactions mediated by the C3b thioester domain for all three poses are primarily electrostatic, but with some Van der Waals contributions as well.

**Figure 7:**
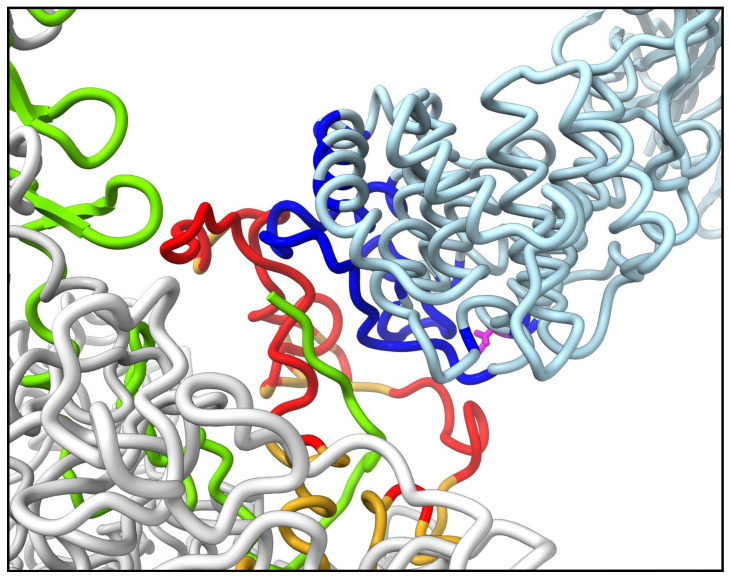
C3b-penton base RGD loop interface. Close-up of the ending coordinates (2 ns) of the MD trajectory for C3b pose 1, viewed as in Figure 6B. Residue Cys1010 of C3b is shown in magenta. Residues in C3b with interaction energies ≤ −1 kcal/mol with penton base chain P2 are colored dark blue. Residues in chain P2 with interaction energies ≤ −1 kcal/mol with C3b are colored red. The remainder of C3b is in light blue; the remainder of chain P2 is in gold; fiber chain F2 is in light green; all other chains are in light gray.

We used gRINN v1.1.0.hf1 to identify specific interacting residues involved in stabilizing interactions between C3b and the penton base RGD loops. The pose 1 MD trajectory ended with the largest number of RGD loop residues involved in favorable interactions with C3b (defined as ≤ −1 kcal/mol). At the ending coordinates (2 ns), 56 residues in the RGD loop of chain P2 interact favorably with 52 residues in the thioester domain (Figure 7).

### 3.4. Structural Model for Factor X Inhibition of IgM and Complement-Mediated Neutralization of HAdv-C5

**Figure 8:**
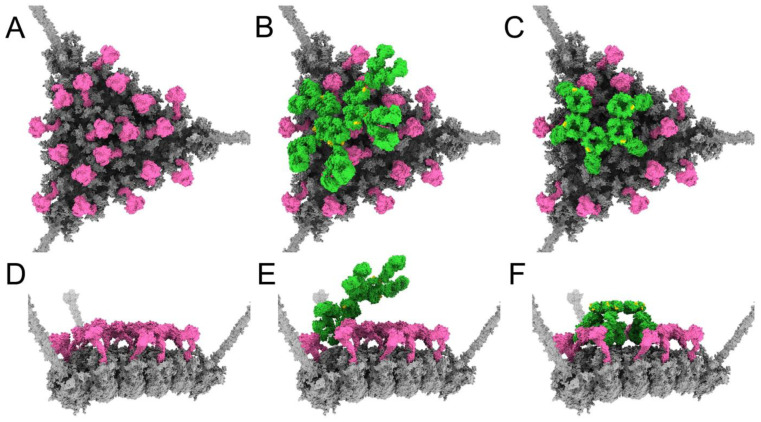
Competition between FX and IgM on adenovirus. (**A**) HAdv-C5 facet (gray) with a copy of FX (pink) bound in the central depression of each of the 18 hexon trimers. (**B**) A planar IgM (green) contacting hexon trimer #1 of the asymmetric unit via the pair of Fab arms counterclockwise from the gap in the Fc platform. The DLPSP binding motifs for C1q on IgM are shown in gold. FX positions are the same as in panel A, but copies clashing with the planar IgM have been removed. (**C**) A bent IgM oriented as in Figure 2. The DLPSP motifs are colored gold. FX positions are the same as in panel A, but copies clashing with the bent IgM have been removed. (**D**) Side view of panel A resulting from a 90° rotation. (**E**) Side view of panel B. (**F**) Side view of panel C.

Having established a rationale for how IgM binding and subsequent complement recruitment leads to neutralization of HAdv-C5, we sought to explain why such neutralization does not occur in the presence of FX. We began by constructing a model where a copy of FX is bound in the center of each hexon trimer on the facet (Figure 8A,D), then investigated the effects of adding planar or bent forms of IgM to the facet. In the presence of FX, we anticipate that copies of FX would occupy most or all of the binding sites on the facet before IgM is able to touch down and interact with the facet. We illustrated this by positioning a planar IgM with a pair of Fab arms hovering over hexon trimer #1 and then removing any copies of FX that clashed with the IgM. The result is a model where an incoming planar IgM has contacted one hexon trimer via a pair of its Fab arms, but nearly all other hexon trimers in the facet are occupied by FX (Figure 8B,E). In this instance, the layer of FX covering the facet surface blocks the remaining Fab arms of IgM from binding to the facet.

It is possible that in some instances, despite the presence of FX, IgM may bind to the facet with all 10 Fab arms. To illustrate this possibility, we placed a bent IgM on the facet in the same position as the model shown in Figure 2, then removed any copies of FX that clashed with the IgM. The resulting model shows a bent IgM with its 10 Fab arms touching down on the facet, and the bent IgM surrounded by FX. The DLPSP motifs on IgM, required for C1q binding, do not appear to be obstructed by the FX molecules (Figure 8C,F). Therefore, it seems possible that the C1 complex could bind the bent IgM under these conditions. However, the limited amount of space around the bent IgM that is not blocked by FX seems to imply that there would be few opportunities for C4b or C3b to bind to the facet under these conditions.

## 4. Discussion

Adenovirus is a promising vector for development of novel gene therapies or oncolytic viruses. However, neutralization by humoral factors of the innate and adaptive arms of the immune system is a significant obstacle to such applications. Understanding the structural bases of interactions between HAdv-C5 and host defense proteins will help to design therapeutic vectors that evade neutralization by the immune system while efficiently fulfilling their targeted function. In this molecular modeling and computational study, we sought to construct and analyze a structural model for IgM and complement-mediated neutralization of FX-binding ablated adenovirus. We were then able to provide a potential mechanistic explanation for the experimental finding that FX inhibits such neutralization.

We began by positioning IgM (with a variety of rotations about its Fc platform) on each of the four hexon trimers within one asymmetric unit of the adenovirus facet in the absence of FX. We then placed complement components C1 and C4b, and finally C3b on the adenovirus facet model. We hypothesized that a C3b molecule present near a vertex of the facet would be able to entangle multiple penton base RGD loops, as has been previously modeled for C4b interacting with adenovirus [49]. Entanglement of the RGD loops would be impactful since integrin binding to the RGD loops is thought to induce a conformational change, or untwisting, of the penton base multimer, which initiates adenovirus uncoating and release of the membrane lytic protein VI [50]. We anticipated that formation of stabilizing interactions between C3b and penton base RGD loops would effectively seal the virus shut.

Indeed, one of our MD simulation vertex models (pose 2) shows C3b interacting with two RGD loops; the associated nonbonded interaction energies are similarly favorable for each of these two loops (−58 and −52 kcal/mol). We interpret this as an indication that C3b can entangle two RGD loops. In addition, there is a strong favorable nonbonded interaction energy between C3b and one fiber chain in this pose, further stabilizing the vertex. In each of the other two vertex models (poses 1 and 3), C3b has a strong favorable interaction with only one RGD loop, but C3b has formed an additional strong interaction with at least one chain of the fiber. Therefore, we revise our hypothesis and propose that formation of multiple stabilizing interactions between C3b and a combination of penton base RGD loops and the fiber can result in neutralization of adenovirus.

When identifying pairs of residues involved in stabilizing interactions between C3b and penton base RGD loops, we found that the interface formed in the pose 1 MD simulation was particularly extensive (Figure 7). It appears that the HAdv-C5 penton base RGD loop is long and flexible enough to adapt and span one side of the C3b thioester domain. We postulate that the intrinsically disordered nature of the RGD loop [51] may allow it to form such extensive interactions with C3b even within relatively short MD simulations.

As our models of an adenovirus facet with IgM, C4b, and C3b indicate, the position of IgM on the facet affects where C3b is most likely to bind. Out of the 16 models generated, one had no C3b bound, and another four had only a single C3b bound (Figure 5). This raises the question of whether limited binding of C3b might influence virus neutralization. In the context of a full virion, each vertex is surrounded by five facets due to the icosahedral symmetry of the capsid. Even if the IgM position on one facet does not lead to C3b at a particular vertex, there are four other facets surrounding that vertex where the IgM position might be different. In other words, there are five chances for a particular vertex to be stabilized by C3b. Consequently, we reason that limited binding of C3b on any one facet would not impede virus neutralization.

To address the key issue of how IgM and complement are able to neutralize Ad5-FX* but not Ad5-WT, we sought to illustrate a structural basis for competition between IgM and FX on the HAdV-C5 facet. In the presence of FX, we anticipated that copies of FX would occupy most or all of their binding sites before IgM could bind via its Fab arms and adopt a bent conformation. We prepared a model in which a planar IgM has managed to position one pair of Fab arms over an unoccupied hexon trimer, but FX is bound to most other hexon trimers in the facet (Figure 8B,E). In this instance, it appears that the dense layer of FX covering the facet surface would block the rest of the IgM Fab arms from binding.

We also considered the possibility that IgM is able to bind via all ten Fab arms and adopt a bent conformation even in the presence of FX. We aimed to determine whether FX inhibition of IgM and complement-mediated neutralization could still occur in this circumstance. In our model with bent IgM surrounded by FX, the DLPSP motifs on IgM, required for C1q binding, do not appear to be obstructed by the FX molecules (Figure 8C,F). Therefore, it appears possible that the C1 complex could still bind to the bent IgM under these conditions. However, in this model limited space remains on the facet surface for C4b or C3b to bind. Previous experiments in mouse naïve serum have shown the amounts of C3b and C4b deposited on the surface, quantified by optical density at 450 nm, are considerably greater for Ad5-FX* than Ad5-WT [24]. Our models of HAdv-C5 with IgM, C1, C4b, and C3b indicate that numerous copies of C4b and C3b can bind per facet in the absence of FX, and only limited copies can bind in the presence of FX.

Ultimately, attempting to add either bent or planar IgM to an FX-occupied HAdv-C5 facet appears to obstruct the process that results in Ad5-FX* being sealed shut by complement. The “planar IgM” facet model provides a plausible explanation for the inability of IgM and complement to inactivate adenovirus in the presence of FX. If IgM cannot contact the facet with most or all of its Fab arms, the bent IgM conformation will not form, and subsequent complement recruitment will not occur. Even if IgM is able to adopt a bent conformation on the facet and complement recruitment does occur, there would be limited space for C4b and C3b. Therefore, in the presence of FX, there would be a low possibility of C3b interacting with capsid proteins at all of the vertices and effectively “sealing” the viral capsid. It would be interesting to test whether or not the isolated FX-GLA domain has the same effect as full length FX. Since the FX-GLA domain binds within the central depression of hexon trimers, it might not interfere with IgM binding. We predict that IgM will bind in a bent conformation even in the presence of FX-GLA, and that this will lead to complement recruitment and viral neutralization. The experimental evaluation of our structural models is a subject of future research. Our modeling and computational study indicates that adenovirus with an FX-binding ablating mutation, such as Ad5-FX*, would allow C1, C4b, and multiple C3b copies to bind to each facet, enabling inactivation of the virus by stabilizing the vertices and preventing release of the virally encoded membrane lytic factor, protein VI. In addition, we show how FX may inhibit IgM and complement-mediated neutralization of adenovirus by preventing C3b from binding near and stabilizing the vertices of the adenovirus capsid.

## Figures and Tables

**Table 1:** Nonbonded interaction energies between C3b, penton base, and fiber. Energies are given for the end of the MD simulations for the 3 poses shown in Figure 6. All energies are in units of kcal/mol and represent the sum of van der Waals and electrostatic interactions between C3b and the indicated chain of penton base or fiber.

C3b Pose	Penton Base Chain P1	Penton Base Chain P2	Penton Base Chain P3	Penton Base Chain P4	Penton Base Chain P5	Fiber Chain F1	Fiber Chain F2	Fiber Chain F3
1	0	−305	0	0	0	0	−198	0
2	−58	−52	0	0	0	−10	0	−173
3	0	0	0	−75	0	9	−123	−47

## Data Availability

The models presented in this study are available on request from the corresponding author.
